# High cholesterol induces apoptosis and autophagy through the ROS-activated AKT/FOXO1 pathway in tendon-derived stem cells

**DOI:** 10.1186/s13287-020-01643-5

**Published:** 2020-03-20

**Authors:** Kaiqun Li, Ye Deng, Ganming Deng, Pengyu Chen, Yutian Wang, Hangtian Wu, Zhiguo Ji, Zilong Yao, Xianrong Zhang, Bin Yu, Kairui Zhang

**Affiliations:** 1 Department of Orthopaedics, Nanfang Hospital, Southern Medical University, No. 1838 Guangzhou Avenue North, Guangzhou, 510515 China; 2 Baoan District People’s Hospital of Shenzhen, Shenzhen, 518100 China; 3 Guangdong Provincial Key Laboratory of Bone and Cartilage Regenerative Medicine, Nanfang Hospital, Southern Medical University, Guangzhou, 510515 China

**Keywords:** Tendinopathy, Tendon-derived stem cells, Cholesterol, Autophagy, Apoptosis, ROS, AKT/FOXO1 pathway

## Abstract

**Background:**

Hypercholesterolemia increases the risk of tendon pain and tendon rupture. Tendon-derived stem cells (TDSCs) play a vital role in the development of tendinopathy. Our previous research found that high cholesterol inhibits tendon-related gene expression in TDSCs. Whether high cholesterol has other biological effects on TDSCs remains unknown.

**Methods:**

TDSCs isolated from female SD rats were exposed to 10 mg/dL cholesterol for 24 h. Then, cell apoptosis was assessed using flow cytometry and fluorescence microscope. RFP-GFP-LC3 adenovirus transfection was used for measuring autophagy. Signaling transduction was measured by immunofluorescence and immunoblotting. In addition, Achilles tendons from ApoE −/− mice fed with a high-fat diet were histologically assessed using HE staining and immunohistochemistry.

**Results:**

In this work, we verified that 10 mg/dL cholesterol suppressed cell proliferation and migration and induced G0/G1 phase arrest. Additionally, cholesterol induced apoptosis and autophagy simultaneously in TDSCs. Apoptosis induction was related to increased expression of cleaved caspase-3 and BAX and decreased expression of Bcl-xL. The occurrence of autophagic flux and accumulation of LC3-II demonstrated the induction of autophagy by cholesterol. Compared with the effects of cholesterol treatment alone, the autophagy inhibitor 3-methyladenine (3-MA) enhanced apoptosis, while the apoptosis inhibitor Z-VAD-FMK diminished cholesterol-induced autophagy. Moreover, cholesterol triggered reactive oxygen species (ROS) generation and activated the AKT/FOXO1 pathway, while the ROS scavenger NAC blocked cholesterol-induced activation of the AKT/FOXO1 pathway. NAC and the FOXO1 inhibitor AS1842856 rescued the apoptosis and autophagy induced by cholesterol. Finally, high cholesterol elevated the expression of cleaved caspase-3, Bax, LC3-II, and FOXO1 in vivo.

**Conclusion:**

The present study indicated that high cholesterol induced apoptosis and autophagy through ROS-activated AKT/FOXO1 signaling in TDSCs, providing new insights into the mechanism of hypercholesterolemia-induced tendinopathy.

**Graphical abstract:**

High cholesterol induces apoptosis and autophagy through the ROS-activated AKT/FOXO1 pathway in tendon-derived stem cells. 
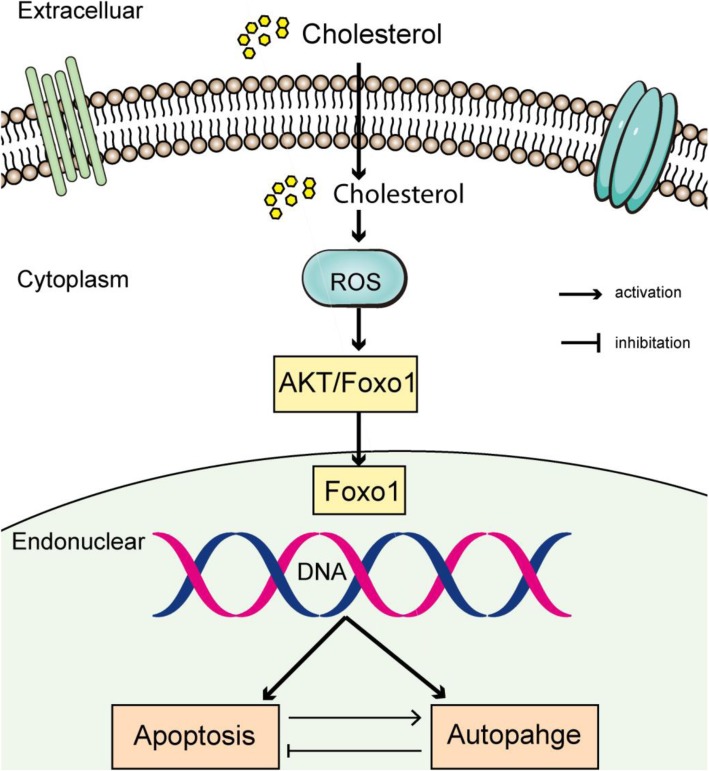

## Introduction

Tendinopathy is a chronic, activity-limiting syndrome characterized by activity-related pain, swelling, or dysfunction. The Achilles tendon is one of the most common sites affected [1]. As a common clinical condition, tendinopathy has not only impacted patient quality of life but also brought a significant disease burden in health care costs [2]. The causes of tendinopathy are multifactorial and are still unclear. Much attention has been given to sports or occupation as risk factors, but there remains an unexplained association of tendinopathy and hypercholesterolemia (familial and nonfamilial), both in active and sedentary populations. Several studies have also suggested that hypercholesterolemia is associated with tendon pathology; therefore, patients with this disease have an increased risk of tendon rupture [3–5]. Furthermore, hypercholesterolemia also has detrimental effects on tendon mechanics [6] and impairs tendon healing [7]. The molecular mechanism underlying high cholesterol-induced tendinopathy remains incompletely understood.

It has been traditionally thought that tendons consist only of tenocytes, the resident cells of the tendons. However, tenocytes are typically quiescent, nondividing cells. Current theories suggesting that a small population of resident stem cells maintain the maintenance and repair of adult tissues have been widely accepted [8]. Recently, tendon-derived stem cells (TDSCs) from human, mouse, and rat tendon tissue have been identified [9, 10]. Unlike static tenocytes, TDSCs are characterized by their multidifferentiation and self-renewing potential. The viability and tenogenic differentiation of TDSCs are closely associated with the maintenance of the tendon microenvironment and the development of tendinopathy [8]. Because tendinopathy is closely correlated with hypercholesterolemia, we found that high cholesterol inhibits tendon-related gene expression in TDSCs [11]. However, the effect of cholesterol on the viability of TDSCs remains unknown.

Apoptosis, a process of programmed cell death, is involved in the maintenance of tissue homeostasis. Excessive apoptosis is associated with degenerative pathologies. Several studies have suggested that apoptosis contributes to the pathology of tendinopathy [12–14]. Apoptosis in tenocytes increases with the severity of induced tendon damage. Autophagy is identified as a preserved self-digestion process that enables cells to recycle unnecessary or dysfunctional components through degradation within lysosomes, also playing an important role in the maintenance of cellular homeostasis [15]. Autophagy has been found to be closely related to apoptosis [16]. In some conditions, autophagy has a protective effect, preventing cells from undergoing apoptosis by promoting cell survival. In others, excessive autophagy induces cell death. Whether high cholesterol induces apoptosis or autophagy in TDSCs and the relationship between apoptosis and autophagy induced by cholesterol remains to be determined.

Reactive oxygen species (ROS), generated from cellular metabolic activities, are the active forms of oxygen [17]. A moderate level of ROS promotes cell proliferation and differentiation, while excessive amounts of ROS induce cell apoptosis or autophagy due to oxidative damage to lipids, proteins, and DNA [18]. We have already found that cholesterol upregulates ROS levels in TDSCs [11]. ROS affect a variety of signaling pathways, including the AKT/FOXO1 signal pathway [19]. The activation of AKT promotes cell growth and proliferation and increases cell resistance to apoptosis in many cell types [20]. As one of the most important substrates of AKT, FOXO1 leads to autophagy and stimulates apoptotic cell death via modulating downstream targets such as apoptosis and autophagy-associated genes [21]. AKT activation mediates the phosphorylation of FOXO1, leading to FOXO1 nuclear exclusion and inactivity, thus suppressing FOXO1-dependent transcription of target genes [22].

Considering the close relationship between cholesterol and tendinopathy and the pivotal role of TDSCs in the pathobiology of tendinopathy, we attempted to evaluate whether cholesterol induces apoptosis and autophagy in TDSCs and investigated the underlying mechanisms, of the induction of apoptosis and autophagy through the ROS-mediated AKT/FOXO1 pathway.

## Materials and methods

### Reagents and antibodies

The reagents used included cholesterol (Sigma, # C4951), collagenase type I (Sigma, #C0130), *N*-acetyl-l-cysteine (NAC, Sigma, #A9165), Z-VAD-FMK (Selleck, #S7023), 3-MA (Selleck, #S2767), and AS1842856 (Selleck, #S8222).

The antibodies used included anti-cleaved caspase-3 (Cell Signaling, #9664), anti-p-FOXO1 (Cell Signaling, #9464), anti-FOXO1 (Huabio, #ET1608), anti-PARP (ZEN Bio, #382828), anti-Bax (ZEN Bio, #382708), anti-Bcl-xL (ZEN Bio, # 382508), anti-LC3B (Proteintech, #18725-1-AP), anti-P62 (Proteintech, #18420–1-AP), anti-AKT (Bimake, # A5023), anti-p-AKT (Bimake, # A5030), and anti-Ki67 (Abcam, #ab15580).

### Cell isolation and cell culture

TDSCs were isolated from the Achilles tendons of female SD rats (6–8 weeks old) as previously described [9]. In this study, all experiments were approved by the Institutional Animal Care and Use Committee of Southern Medical University (Guangzhou, China). After euthanasia, the Achilles tendons were separated from both limbs of each rat. The midsubstance of the Achilles tendons was stripped off the tendon sheath and cut into small pieces. Then, the samples were washed with sterile PBS and digested with collagenase type I (3 mg/mL) for 2 h at 37 °C. After passing through a 70-mm cell strainer, the released cells were centrifuged at 2000 rpm for 15 min and seeded in culture plates. The isolated cells were cultured in medium (LG-DMEM, Gibco) with 20% fetal bovine serum (FBS, Gibco), 1% penicillin/streptomycin, and 100 mM 2-mercaptoethanol (Gibco) for 8–10 days at 37 °C and 5% CO_2_ to form colonies. When the primary cells reached 90% confluence, the cells were harvested with trypsin/EDTA and expanded in a second passage. Then, the cells were cryopreserved for subsequent experiments. After thawing, the third-passage cells were used for the experiments in this study. The culture medium was changed every 3 days throughout the experiments.

### Cell viability assay

TDSC proliferation was evaluated using the Cell Counting Kit-8 assay (CCK-8, Dojindo Molecular Technologies, # KL640). In brief, TDSCs were seeded in 96-well plates at a concentration of 1000 cells per well and then cultured in medium with 0, 1, 10, or 100 mg/dL cholesterol for 1, 3, or 5 days. TDSC proliferation was analyzed by measuring the absorbance at 450 nm using a microplate reader according to the manufacturer’s protocol.

### Wound healing assay

A wound healing assay was carried out to determine the cell migration of TDSCs. TDSCs were seeded in 6-well plates at a density of 1 × 10^5^ cells/dish. When the TDSCs reached 100% confluence, a sterile 200-μL pipette tip was used to make a straight scratch in the cell monolayer. Subsequently, PBS was used to gently wash the scratched areas twice. TDSCs were then cultured in the abovementioned medium with or without cholesterol (10 mg/dL) at 37 °C. After the TDSCs were scratched, pictures of the entire scratched areas in each group were acquired at 0, 12, 24, 36, 48, and 60 h using a phase-contrast microscope (Olympus Corporation, Tokyo, Japan). The migration rate was evaluated using the equation ((the recovered gap area/the initial scratch area) × 100%).

### Cell cycle assay

TDSCs were seeded in 6-well plates at a density of 1 × 10^6^ cells/dish and cultured in the abovementioned medium with or without cholesterol (10 mg/dL) for 24 h. Based on the manufacturer’s protocol for the cell cycle assay kit (KeyGen Biotech Co, Nanjing, China), the cells were harvested and then fixed with cold 70% ethanol at 4 °C overnight. Next, the TDSCs were washed twice with PBS and incubated in RNase A at 37 °C for 30 min. Then, the cells were stained with propidium iodide at 4 °C for 30 min. Finally, 1 × 10^4^ cells in each group were tested for cell cycle assay by a flow cytometer (BD Biosciences, San Jose, CA, USA).

### Apoptosis analysis

An annexin V-FITC apoptosis detection kit (Beyotime, Beijing, China) was used to analyze the apoptotic cells. TDSCs were plated in 6-well plates at a density of 1 × 10^6^ cells/dish and incubated in the abovementioned medium with or without cholesterol (10 mg/dL) for 24 h. Then, the cells were collected, mixed with 1× binding buffer, and stained with FITC-conjugated annexin V and PI for 15 min in the dark at room temperature. The apoptotic cells were measured by a flow cytometer and analyzed by the software supplied with the instrument.

### TUNEL assay

A TUNEL assay kit (Beyotime, Beijing, China) was used to detect DNA fragments in apoptotic cells. Briefly, TDSCs were seeded on glass coverslips in a 12-well plate at a density of 3 × 10^4^ cells/dish and treated with or without cholesterol (10 mg/dL) for 24 h. After treatment, the cells on the glass coverslips were washed twice with PBS, fixed with 4% formaldehyde for 30 min, and perforated with 0.5% Triton X-100 for 5 min. Then, the cells were incubated with TUNEL reaction mixture for 1 h in the dark at room temperature, and the nuclei were stained with DAPI. Finally, TUNEL-positive TDSCs were observed under a fluorescence microscope (Olympus, Japan) and counted.

### Autophagic flux analysis

Autophagic flux in TDSCs was detected by using the mRFP-GFP-LC3 adenovirus (Hanbio, China). After plating the cells in a 24-well plate at a density of 1 × 10^4^ cells/dish and incubating with mRFP-GFP-LC3 adenovirus for 24 h, the cells were treated with or without cholesterol (10 mg/dL) for 24 h. Autophagic flux was observed under an inverted fluorescent microscope (Olympus, Japan). The yellow puncta indicated autophagosomes, and the red puncta indicated autolysosomes.

### Western blotting

Western blotting was performed based on a previously described protocol [11]. After the indicated stimulation, proteins from TDSCs were extracted with RIPA buffer containing PMSF, and protease and phosphatase inhibitor cocktails. Then, 30 μg of protein from each sample was separated by SDS-PAGE and transferred to PVDF membranes. The membranes were blocked in 5% BSA for 1 h at room temperature and then incubated with primary antibodies at 4 °C overnight, followed by peroxidase-conjugated secondary antibody for 1 h at room temperature. Protein expression was measured by using a chemiluminescence kit (Millipore, Plano, TX, USA) and analyzed by using ImageJ.

### Measurement of intracellular ROS levels

Intracellular ROS levels were detected by using a reactive oxygen species assay kit according to the manufacturer’s protocol. After treatment with or without cholesterol, TDSCs were incubated with 10 μM DCFH-DA at 37 °C in the dark for 30 min and then analyzed by a flow cytometer.

### Immunofluorescence staining

Immunofluorescence staining was performed as previously described. The cells were seeded on glass coverslips in 12-well plates at a density of 10^4^ cells/dish and subjected to different treatments for 24 h. After washing three times with PBS, the cells were fixed with 4% formaldehyde and perforated with 0.5% Triton X-100. Then, the cells were blocked with 5% BSA and incubated with primary antibodies at 4 °C overnight and with FITC-conjugated goat antibody. The nuclei were stained with DAPI. Finally, the images were acquired under a fluorescence microscope.

### Animal experimental design

As a widely used hypercholesterolemic animal model, six male C57BL/6 mice that were deficient for apolipoprotein E (APOE) and fed a high-fat diet were used as the hypercholesterolemia group. Correspondently, six male C57BL/6 mice represented the control group. Both groups of mice were purchased from the Laboratory Animal Center of Southern Medical University, Guangzhou, at 10 weeks old and were fed until 10 months old. After euthanasia, the Achilles tendons were harvested for HE staining and immunostaining. Animals were housed under a 12:12 h light/dark cycle at 22–24 °C.

### HE staining and immunohistochemistry

After fixation with 4% formalin for 24 h, the Achilles tendons were embedded in paraffin and cut into 6-μm sections. The sections were stained with HE according to a standard protocol. The Bonar scoring system was utilized for histological analysis as previously described [11]. For immunohistochemical detection of cleaved caspase-3, Bax, LC3B, p62, and FOXO1, the sample sections were incubated with the corresponding primary antibody based on a standard protocol. The positive cell rate was calculated using ImageJ software.

### Statistical analysis

The experimental data are presented as the mean ± standard error (SEM) from at least three independent experiments. A two-tailed Student’s *t* test was used to calculate the difference between two groups. One-way ANOVA was performed to analyze more than two groups, followed by Dunnett’s test. A *P* value < 0.05 was deemed to be significant. SPSS 20 software (IBM, NY, USA) was utilized in all statistical analyses.

## Results

### Cholesterol inhibits the proliferation and migration of TDSCs and induces G0/G1 phase arrest

**Fig. 1:**
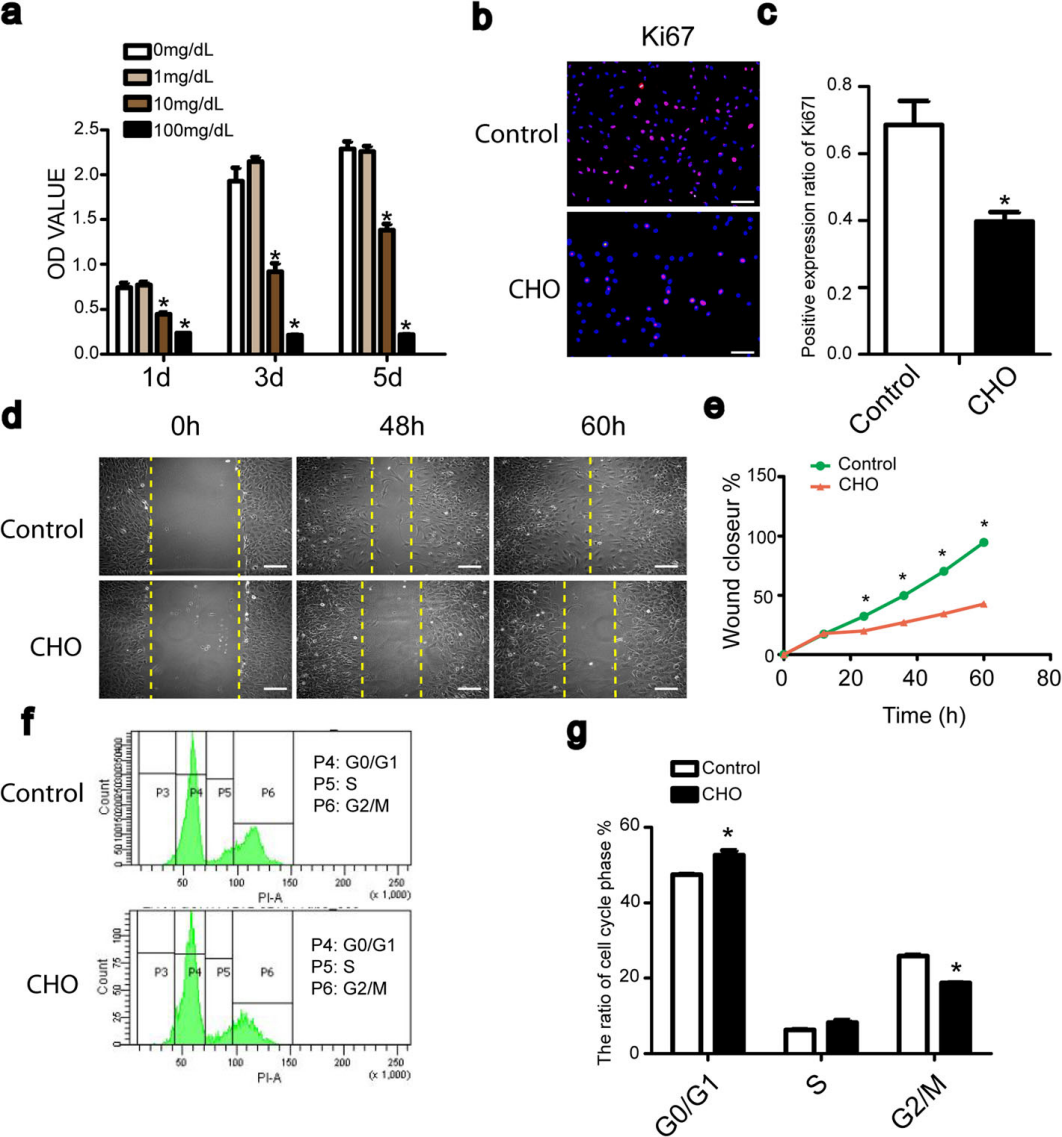
Cholesterol inhibits the proliferation and migration of TDSCs and induced G0/G1 phase arrest. **a** Cell viability was assessed by CCK-8 assay after treatment with various concentrations of cholesterol for different periods of time. **b**, **c** Cells were treated with 10 mg/dL cholesterol for 24 h. Ki67 expression was analyzed by immunofluorescence. Bar, 50 μm. **d**, **e** After treatment with 10 mg/dL cholesterol, the migratory capacity of TDSCs was assessed by wound healing assay. Bar, 100 μm. **f**, **g** Cells were treated with 10 mg/dL cholesterol for 24 h. The percentage of the cell population at G0/G1, S, and G2/M was analyzed by flow cytometry. All quantitative data are expressed as the means ± SEM of the results from three independent experiments. *p < 0.05 versus control. CHO, cholesterol

To evaluate the effect of cholesterol on the proliferation of TDSCs, cells were exposed to various concentrations of cholesterol for 1, 3, or 5 days, and cell viability was measured using the CCK-8 assay. The experimental data showed that the inhibition was significant in the 10 and 100 mg/dL cholesterol groups at 1, 3, and 5 days (Fig. 1a). Thus, TDSCs were treated with 10 mg/dL cholesterol for 1 day in the subsequent experiment. As a proliferation marker, the Ki67-positive ratio of TDSCs was also significantly reduced, as shown by immunofluorescence staining (Fig. 1b, c). These results suggest that 10 mg/dL cholesterol inhibits the viability of TDSCs. To investigate whether cholesterol inhibited cell viability by inducing cell cycle arrest, the distribution of the cell cycle was determined in TDSCs treated with cholesterol. Figure 1 f and g show that cholesterol increased the number of cells in G0/G1 phase and reduced the number of cells in G2/M and S phases in TDSCs. Next, we performed a wound healing assay to assess whether cholesterol inhibits the migration of TDSCs. Microscopy and quantitative analyses indicated that the wound healing capacity in cholesterol-treated TDSCs was significantly impaired beginning at 24 h compared with that of the control cells (Fig. 1d, e).

### Cholesterol induces apoptosis in TDSCs

**Fig. 2:**
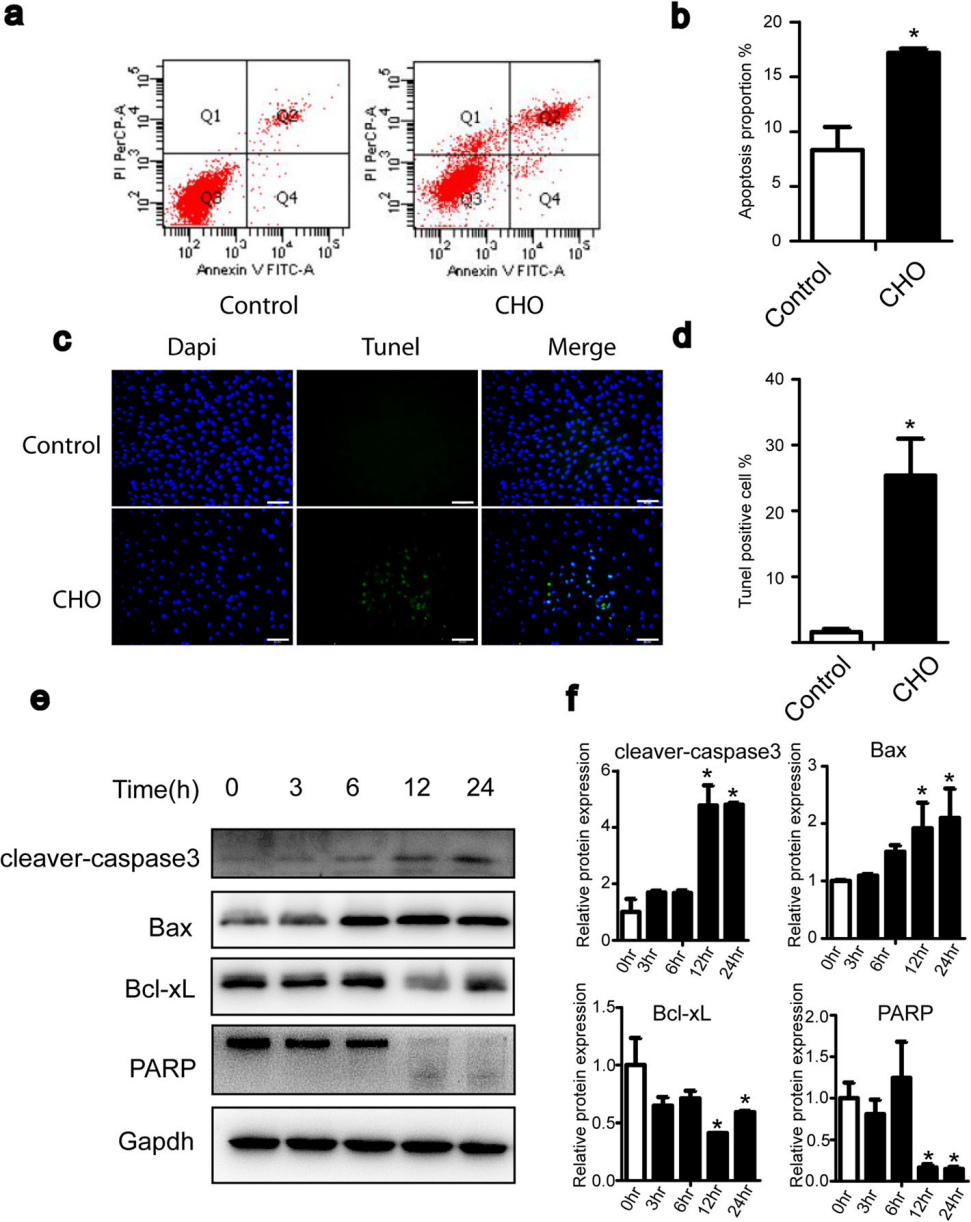
Cholesterol induces apoptosis in TDSCs. **a**, **b** Cells were treated with 10 mg/dL cholesterol for 24 h and analyzed using annexin V-FITC flow cytometry. **c**, **d** DNA fragmentation was detected by TUNEL staining and observed by fluorescence microscopy. **e**, **f** Cells were incubated with cholesterol (10 mg/dL) for different times. The apoptosis-related proteins cleaved caspase-3, Bax, Bcl-xL, and PARP were analyzed by western blotting. All quantitative data are expressed as the means ± SEM of the results from three independent experiments. *p < 0.05 versus control. CHO, cholesterol

To examine whether the cholesterol-mediated inhibition of proliferation in TDSCs was related to the induction of apoptotic cell death, TDSCs were exposed to 10 mg/dL cholesterol for 24 h and then analyzed by flow cytometry using an annexin V-FITC apoptosis detection kit (Fig. 2). As shown in Fig. 2a and b, cholesterol treatment significantly induced apoptotic cells. Furthermore, the TUNEL assay was used to detect DNA fragmentation in apoptotic cells. The results demonstrated that treatment with cholesterol led to an increase in apoptotic TDSCs (Fig. 2c, d). Next, we assessed apoptosis-related proteins using western blotting to investigate the possible mechanism of cholesterol-induced apoptosis. As shown in Fig. 2e and f, treatment with cholesterol for 12 and 24 h upregulated cleaved caspase-3 protein levels and downregulated the expression of poly (ADPribose) polymerase (PARP), which suggests that PARP was cleaved. Bcl-2 family members are known to be involved in the mitochondrial-mediated apoptosis pathway; antiapoptotic proteins, including BCL-xL, and proapoptotic proteins, such as BAX, are the most important regulators of apoptosis processes [23]. Thus, we investigated the expression of the key members of the Bcl-2 family, Bax and Bcl-xL. As shown in Fig. 2e and f, treatment with cholesterol for 12 and 24 h upregulated the Bax protein level and downregulated the expression level of Bcl-xL in TDSCs. These results indicate that cholesterol induces mitochondrial-mediated pathways.

**Fig. 3:**
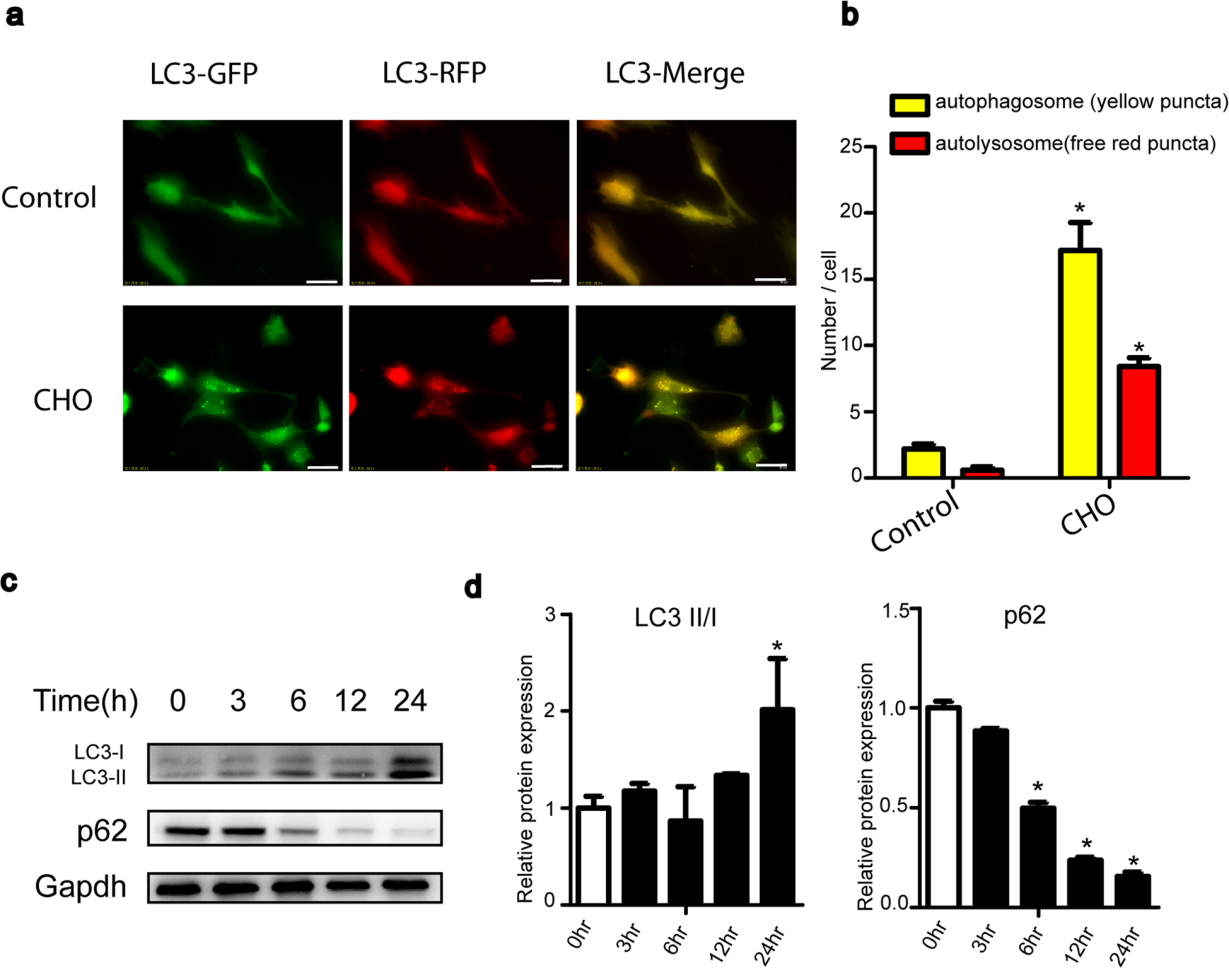
Cholesterol induces autophagy in TDSCs. **a**, **b** Cells were transfected with the mRFP-GFP-LC3 vector for 24 h and then treated with cholesterol (10 mg/dL) for 24 h. Representative images of fluorescent LC3 puncta are shown. The mean number of yellow puncta representing autophagosomes and the mean number of red puncta representing autolysosomes are plotted. Bar, 50 μm. **c**, **d** Cells were incubated with cholesterol (10 mg/dL) for different times. The autophagy-related proteins LC3-II and p62 were analyzed by western blotting. All quantitative data are expressed as the means ± SEM of the results from three independent experiments. *p < 0.05 versus control. CHO, cholesterol

### Cholesterol induces autophagy in TDSCs

As autophagy is closely linked to apoptosis, we explored whether cholesterol also induces autophagy in TDSCs. We used western blotting to analyze the expression of LC3-II, a general autophagosomal marker. The LC3-I to LC3-II conversion markedly increased, as shown in Fig. 3c and d. Our experimental data revealed that cholesterol treatment decreased protein expression of p62, which is an adaptor protein mediating the ubiquitination and degradation of LC3 (Fig. 3c, d). The clearance of p62 further verified the cholesterol-induced autophagy. To further confirm the autophagy induced by cholesterol, TDSCs were transfected with mRFP-GFP-LC3 adenovirus to detect autophagy flux. As shown in Fig. 3a and b, the number of yellow and red puncta both increased markedly in the merged pictures. The yellow and red puncta in the merged images represent autophagosomes and autolysosomes, respectively.

### Inhibition of autophagy enhances apoptosis, while repression of apoptosis diminishes autophagy

**Fig. 4:**
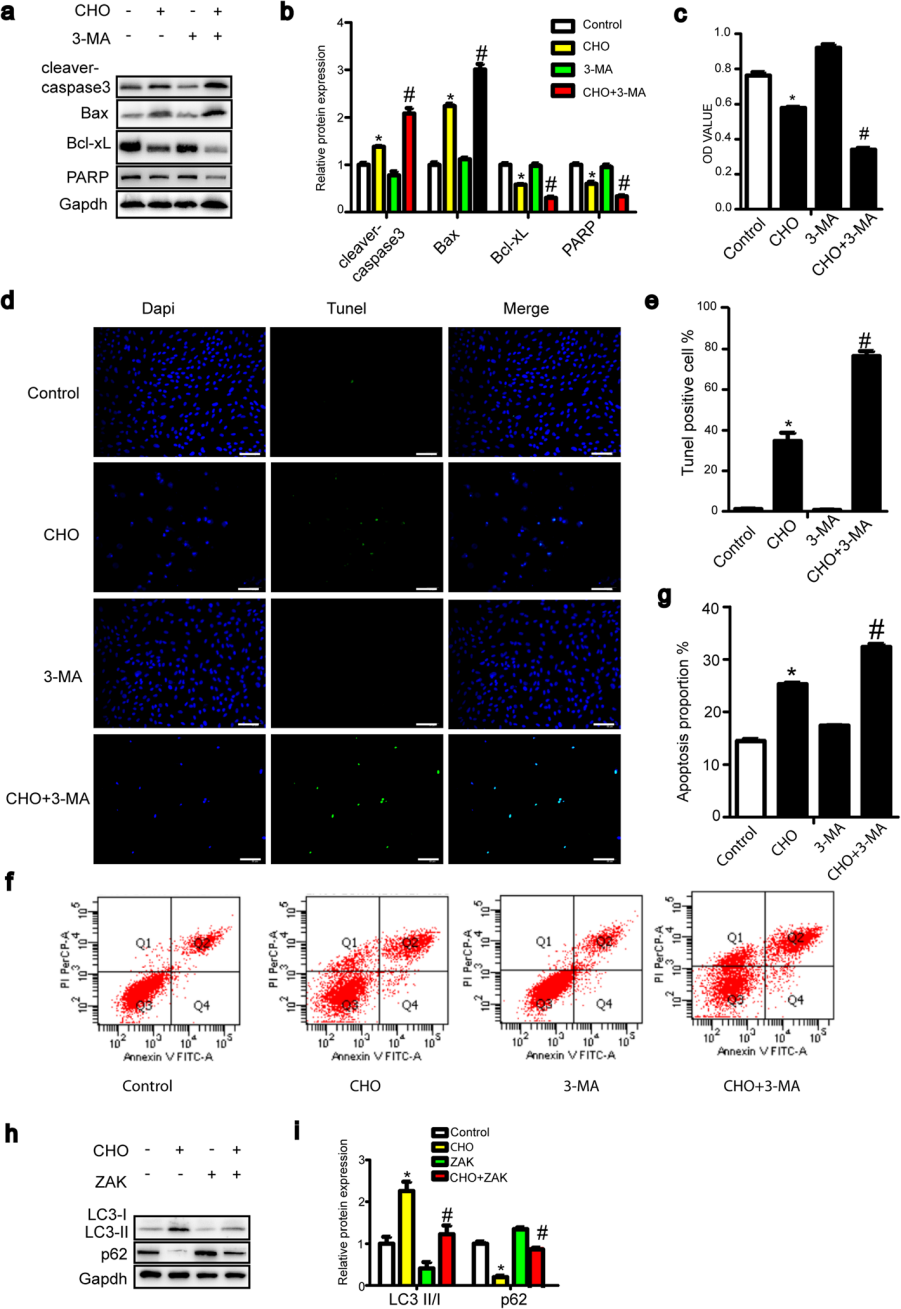
Inhibition of autophagy enhances apoptosis, while suppression of apoptosis diminishes autophagy. **a**–**g** TDSCs were pretreated with 3-MA (1 mM, 1 h) before incubation with 10 mg/dL cholesterol and 3-MA for 24 h. Apoptosis-related proteins (cleaved caspase-3, Bax, Bcl-xL, and PARP) (**a**, **b**) were analyzed by western blotting. The apoptosis proportion was evaluated by TUNEL staining (**g**, **h**, bar, 50 um) and annexin V-FITC flow cytometry (**d**–**g**). Cell viability was assessed by CCK-8 assay (**c**). **h**, **i** TDSCs were pretreated with Z-VAD-FMK (20 μM, 1 h) before incubation with 10 mg/dL cholesterol and Z-VAD-FMK for 24 h. Autophagy-related proteins (LC3-II and p62) were analyzed by western blotting. All quantitative data are expressed as the means ± SEM of the results from three independent experiments. *p < 0.05 versus control, ^#^*p* < 0.05 versus CHO. CHO, cholesterol; ZAK, Z-VAD-FMK

Substantial evidence revealed that complex feedback loops and crosstalk exist between apoptotic and autophagic signaling pathways. To clarify the interaction between autophagy and apoptosis, TDSCs were pretreated with the autophagy inhibitor 3-MA or the apoptosis inhibitor Z-VAD-FMK. As shown in Supplementary Fig. 1a and c, 3-MA successfully inhibited cholesterol-induced autophagy. A significant increase in the protein levels of cleaved BAX and caspase 3 and a marked decrease in the protein levels of Bcl-xL and PARP in TDSCs treated with cholesterol and 3-MA were observed compared with those of cholesterol treatment alone (Fig. 4a, b). Moreover, the numbers of apoptotic TDSCs were significantly increased (Fig. 4d, f). In addition, the viability of TDSCs decreased to a greater degree in the presence of 3-MA (Fig. 4c). These results revealed that autophagy was indispensable for the protection of TDSCs from cholesterol-induced apoptotic death. The level of cholesterol-induced autophagy was also examined in the presence of Z-VAD-FMK. Treatment with Z-VAD-FMK led to a partial reduction in cholesterol-induced autophagy (Fig. 4h, i), suggesting that cholesterol-induced autophagy depended on apoptosis. These results suggest that inhibition of autophagy enhances apoptosis, while repression of apoptosis diminishes autophagy.

### Cholesterol-induced ROS initiate apoptosis and autophagy in TDSCs

**Fig. 5:**
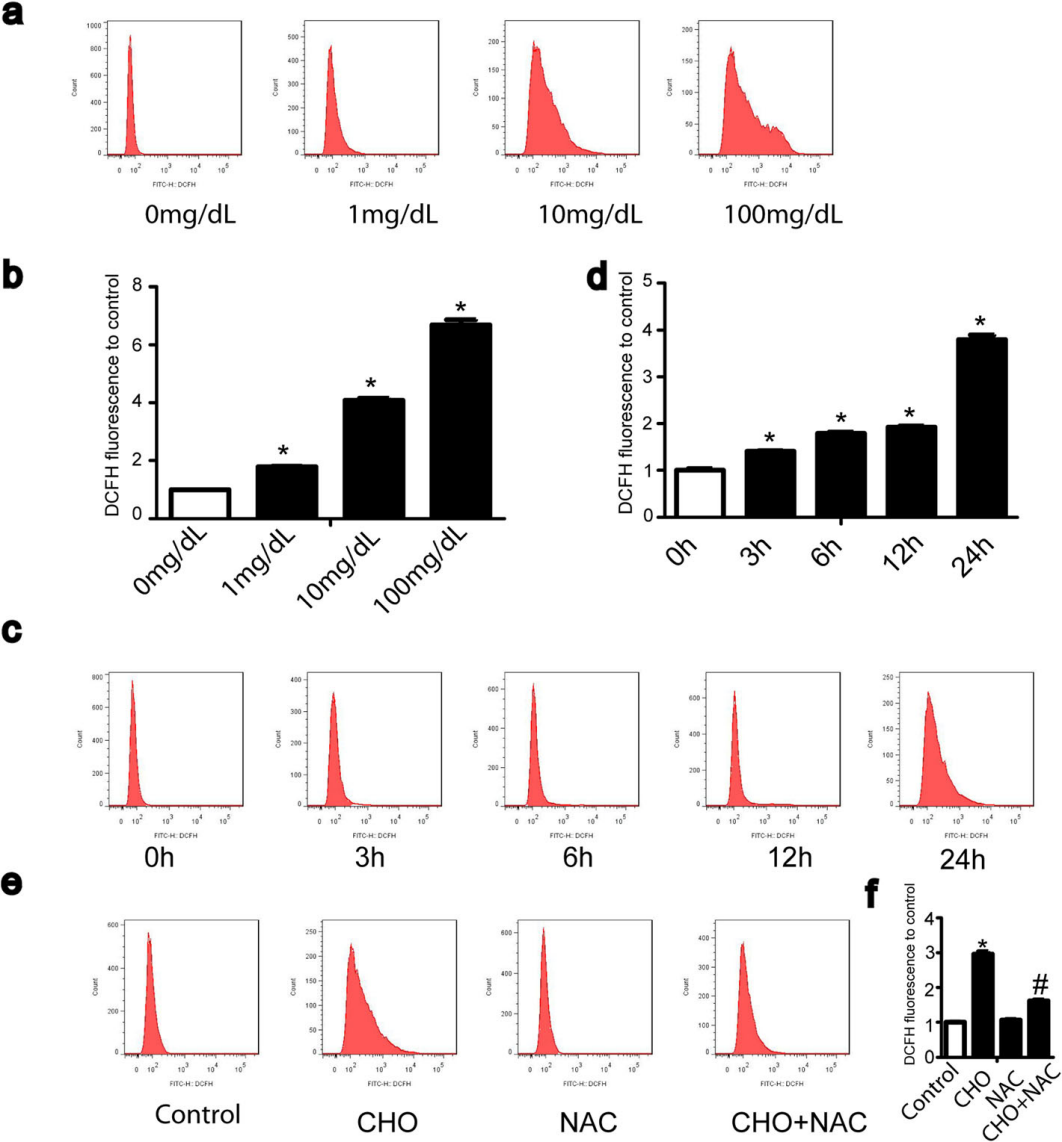
Cholesterol stimulates cellular ROS generation in TDSCs. **a**, **b** TDSCs were treated with the indicated concentrations of cholesterol for 24 h, followed by detection of ROS levels by flow cytometry. **c**, **d** TDSCs were treated with cholesterol (10 mg/dL) for different periods of time, followed by detection of ROS levels by flow cytometry. **e**, **f** TDSCs were pretreated with NAC (5 mM, 1 h) before incubation with 10 mg/dL cholesterol and NAC for 24 h, followed by detection of ROS levels by flow cytometry. All quantitative data are expressed as the means ± SEM of the results from three independent experiments. *p < 0.05 versus control

**Fig. 6:**
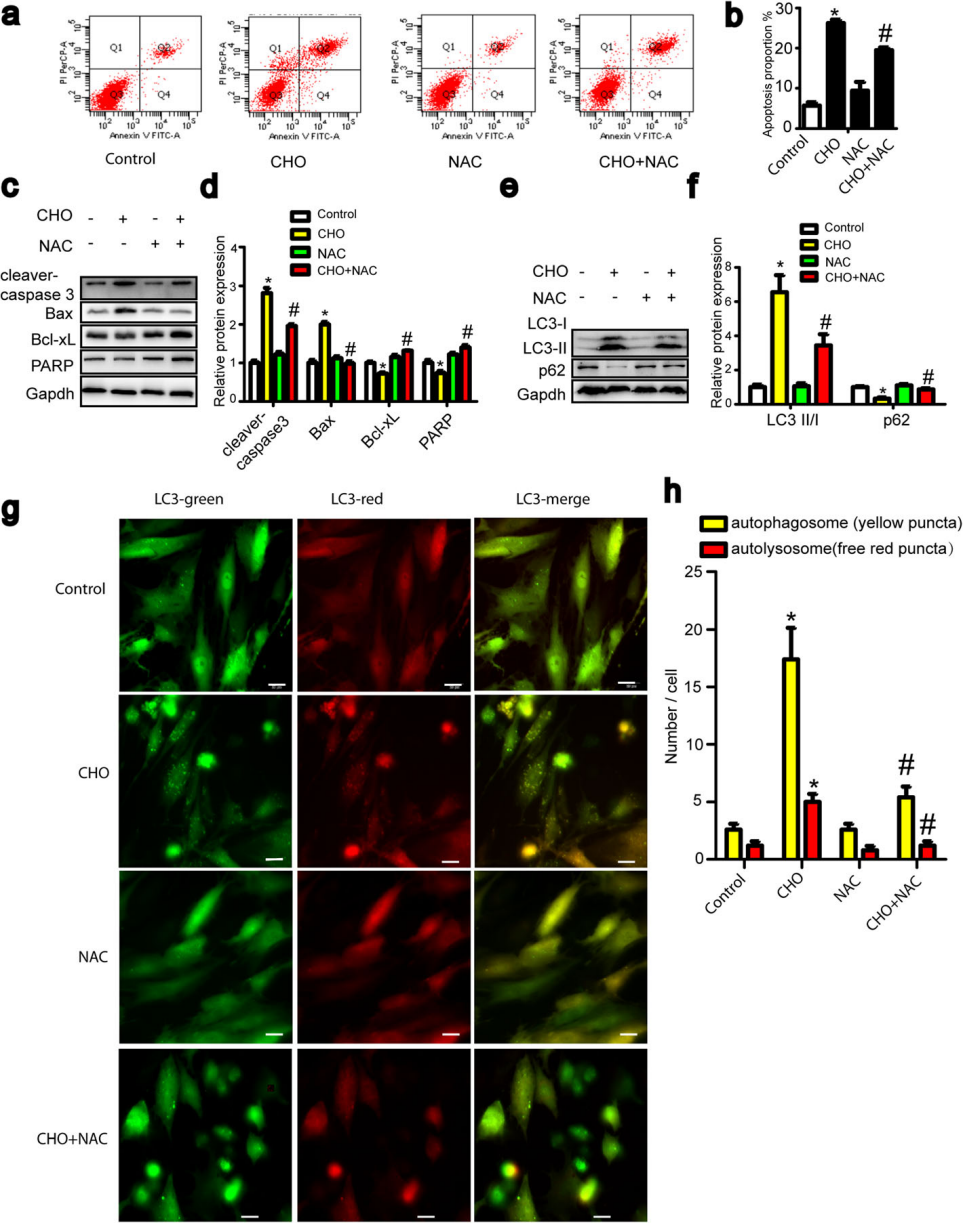
Cholesterol-induced ROS initiate apoptosis and autophagy in TDSCs. **a**–**f** TDSCs were pretreated with NAC (5 mM, 1 h) before incubation with 10 mg/dL cholesterol and NAC for 24 h. The apoptosis proportion was evaluated by annexin V-FITC flow cytometry (**a**, **b**). Apoptosis-related proteins (cleaved caspase-3, Bax, Bcl-xL, and PARP) (**c**, **d**) and autophagy-related proteins (LC3-II and p62) (**e**, **f**) were analyzed by western blotting. **g**, **h** Cells were transfected with the mRFP-GFP-LC3 vector for 24 h and then pretreated with NAC (5 mM, 1 h) before incubation with 10 mg/dL cholesterol and NAC for 24 h. Representative images of fluorescent LC3 puncta are shown. Bar, 20 μm. All quantitative data are expressed as the means ± SEM of the results from three independent experiments. *p < 0.05 versus control, ^#^*p* < 0.05 versus CHO. CHO, cholesterol

ROS usually act as important regulators in apoptosis and autophagy. Our previous report demonstrated that cholesterol upregulated ROS levels and the expression of the oxidative stress-related proteins CAT and NOX4 in TDSCs [11]. Furthermore, we determined the ROS levels in TDSCs treated with cholesterol in dose- and time-dependent manners. The ROS level increased in dose- and time-dependent manners in response to cholesterol stimulation, as shown in Fig. 5a and c. Then, we used the ROS scavenger NAC to investigate the role of ROS in cholesterol-induced cell apoptosis and autophagy. ROS levels were markedly eliminated by NAC (Fig. 5e). Fewer autophagosomes and autolysosomes were observed in cells pretreated with NAC (Fig. 6g, h). NAC also rescued the cholesterol-induced apoptotic proportions (Fig. 6a, b). Furthermore, NAC suppressed the cholesterol-induced activation of apoptosis and autophagy-related proteins (Fig. 6c, e). These results revealed that ROS initiated cholesterol-induced apoptosis and autophagy.

### Cholesterol-induced ROS initiate apoptosis and autophagy in TDSCs through the AKT/FOXO1 pathway

**Fig. 7:**
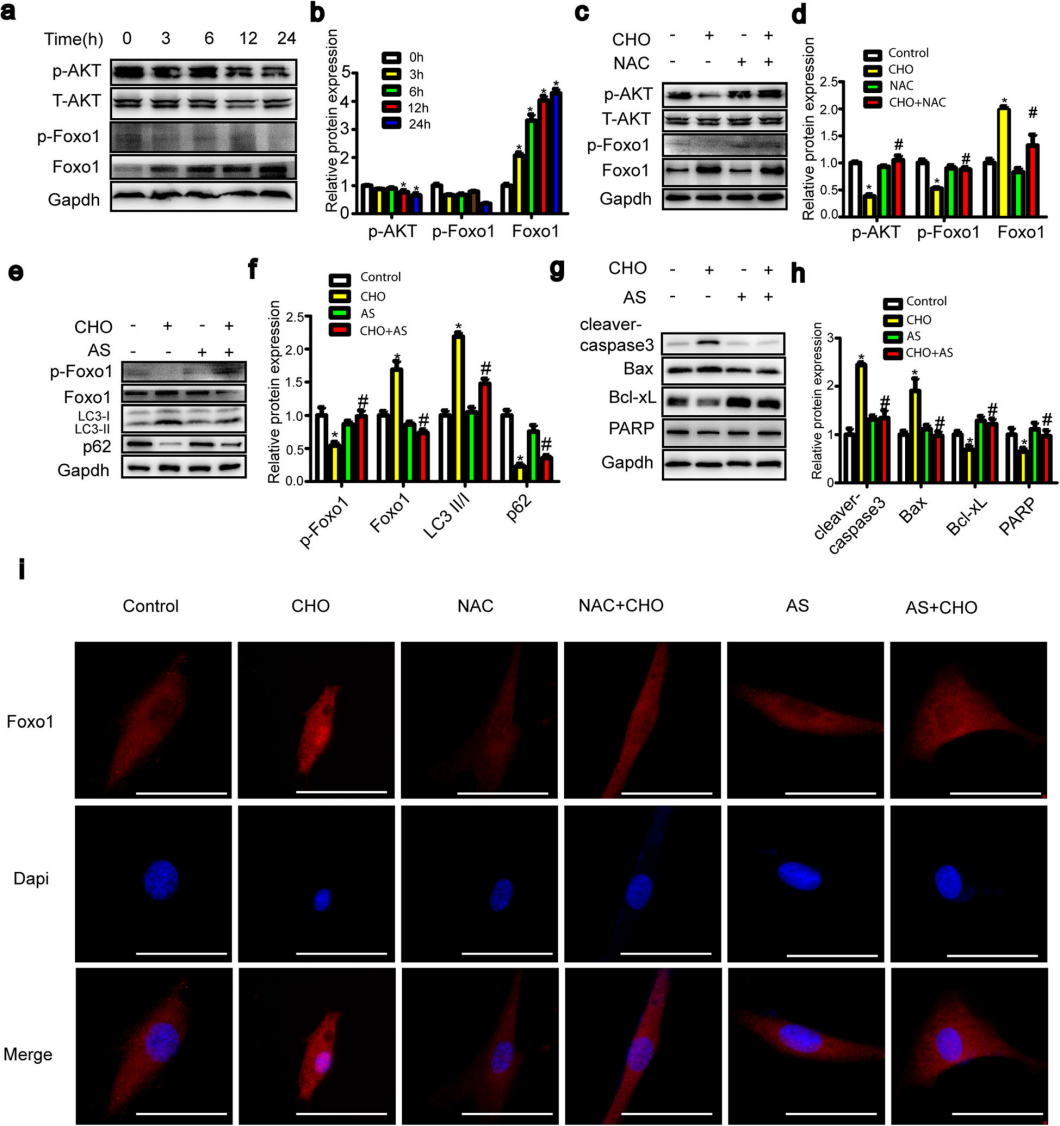
Cholesterol-induced ROS initiate apoptosis and autophagy in TDSCs through the AKT/FOXO1 pathway. **a**, **b** TDSCs were treated with cholesterol (10 mg/dL) for different periods of time. Levels of p-AKT, T-AKT, p-FOXO1, and FOXO1 were analyzed by western blotting. **c**, **d** TDSCs were pretreated with NAC (5 mM, 1 h) before incubation with 10 mg/dL cholesterol and NAC for 24 h. Levels of p-AKT, T-AKT, p-FOXO1, and FOXO1 were analyzed by western blotting. **e**–**h** TDSCs were pretreated with AS1842856 (10 μM, 1 h) before incubation with 10 mg/dL cholesterol and AS1842856 for 24 h. Levels of p-FOXO1, FOXO1, LC3-II, p62, cleaved caspase-3, Bax, Bcl-xL, and PARP were analyzed by western blotting. **i** Representative fluorescent images of TDSCs pretreated with or without NAC (5 mM, 1 h) and AS1842856 (10 μM, 1 h), incubated with cholesterol for 24 h and stained with FoxO1 antibody. FoxO1 proteins are shown in red, nuclei were stained with DAPI (in blue), and the bar is 50 μm. All quantitative data are expressed as the means ± SEM of the results from three independent experiments. *p < 0.05 versus control, ^#^*p* < 0.05 versus CHO. CHO, cholesterol

The AKT protein has been reported to promote cell proliferation in many cell types. We thus used western blotting to detect the expression of total and phosphorylated levels of AKT protein under cholesterol stimulation. Figure 7 a and b show that the phosphorylation of AKT was inhibited by cholesterol. In addition, we found that the levels of FoxO1 were increased in a time-dependent manner in cholesterol-treated TDSCs, from 3 to 24 h. Moreover, the levels of phospho-FoxO1 were markedly decreased compared with those of the controls. As shown in Fig. 7i, exposure of TDSCs to cholesterol resulted in noticeable nuclear translocation of FoxO1. To determine whether the FOXO1 transcription factor affects the ability of cholesterol to induce apoptosis and autophagy, we detected alterations in apoptosis and autophagy-related proteins in the presence of the FOXO1 inhibitor AS1842856 using western blotting. As illustrated in Fig. 7e and g, AS1842856 significantly reversed cholesterol-induced apoptosis and autophagy. Furthermore, we assessed the effects of ROS production on the AKT/FOXO1 pathways. The results showed that combination treatment with cholesterol and NAC increased FOXO1 and AKT phosphorylation and decreased the expression of FOXO1 (Fig. 7c, d) and the accumulation of FOXO1 in the nucleus compared with that of cholesterol treatment alone. These results suggest that ROS may be an important factor upstream of AKT/FOXO1 that initiates cholesterol-induced apoptosis and autophagy.

### High cholesterol induces apoptosis and autophagy in vivo

**Fig. 8:**
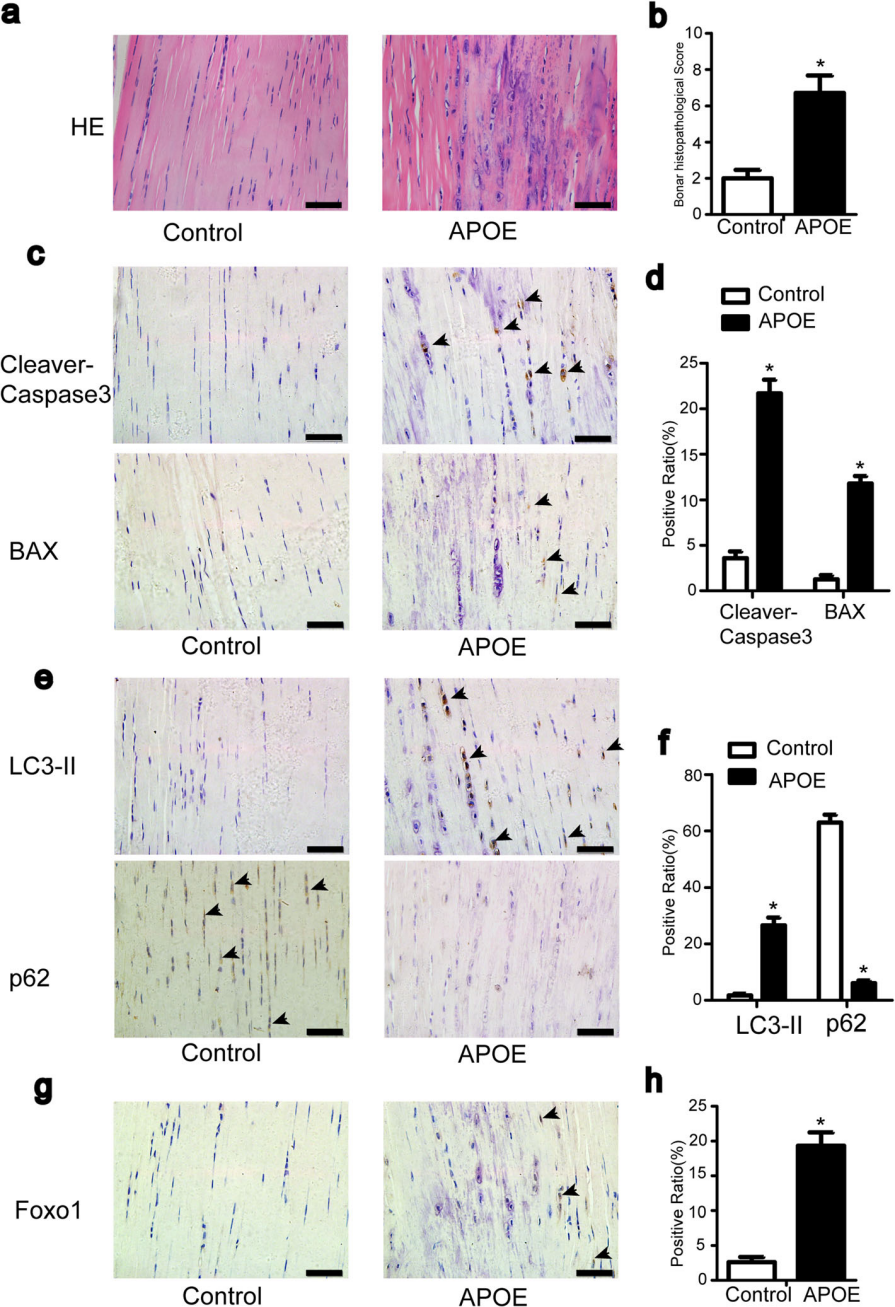
High cholesterol induces apoptosis and autophagy in vivo. Six C57BL/6 mice (10 months old, male) fed a normal diet and six APOE mice (10 months old, male) fed a high-fat diet representing the control and hypercholesterolemic groups, respectively. **a**, **b** Histological alterations were evaluated with HE staining. **c**–**h** The indicated proteins were detected by immunohistochemistry. All quantitative data are expressed as the means ± SEM. *p < 0.05 versus control. Bar, 50 μm. Arrow indicates positive stained cell

To simulate hypercholesterolemia in vivo, we used ApoE −/− mice (10 months old, male) as the hypercholesterolemic group, while C57BL/6 mice (10 months old, male) were the control group. As previously reported [11], the Achilles tendon showed significant histopathological abnormalities in the hypercholesterolemic group. In contrast to the typically elongated shape, the shape of the nuclei of some tenocytes became round, similar to a chondroid change (Fig. 8a). Compared with the scores for normal tendons, the Bonar score for Achilles tendons in the hypercholesterolemic group was much higher (Fig. 8b). Immunohistochemical analysis confirmed the increased expression of cleaved caspase-3, Bax, LC3-II, and FOXO1 and decreased expression of p62 in the hypercholesterolemic group (Fig. 8c, e, g). These results indicated that cholesterol induces apoptosis and autophagy in vivo. We reported that CAT and NOX4 protein expression were also significantly increased in the hypercholesterolemic group [11]. Overwhelming evidence has elucidated that NOX4 is a source of ROS, and CAT is an antioxidant protein. Based on these results, we surmised that high cholesterol induces apoptosis and autophagy via the ROS-mediated FOXO1 pathway.

## Discussion

Clinical studies have suggested an association between hypercholesterolemia and tendon injuries or chronic tendon pain [24]. We previously reported that hypercholesterolemia induces tendinopathy in Achilles tendons of ApoE −/− mice [11]. The precise mechanisms underlying this association are not yet known. TDSCs play a pivotal role in tendon physiology and pathobiology in tendinopathy. High cholesterol inhibits tendon-related gene expression in TDSCs. Whether high cholesterol has other biological effects on TDSCs remains unknown. In this study, we found that high cholesterol induced apoptosis and autophagy via the ROS-mediated FOXO1 pathway in TDSCs, suggesting a new mechanism underlying hypercholesterolemia-induced tendinopathy.

Apoptosis is an important component in the control of cell proliferation and maintaining cellular homeostasis. Increased tenocyte apoptosis is believed to be the underlying mechanism responsible for tendinopathy. Previous studies have shown a direct correlation between the severity of damage-induced, increased apoptotic activity, and the inhibition of remodeling genes [25, 26], suggesting that apoptosis may be implicated in the pathogenesis of tendinopathy. However, previous studies have mainly focused on the proapoptotic effect of repetitive stress and overuse on tenocytes. In this study, we investigated whether high cholesterol has a similar proapoptotic effect on TDSCs. Our data suggested that high cholesterol inhibits the proliferation and migration of TDSCs and induces G0/G1 phase arrest, indicating that the viability of TDSCs is seriously impaired by high cholesterol. We found that high cholesterol induced an increase in apoptotic cells and DNA fragmentation in TDSCs. We hypothesize that this process has been linked to lipotoxicity. The mitochondrial or intrinsic pathway is a key pathway related to apoptosis.

After being triggered by different factors, Bax protein migrates to the surface of the mitochondrion and inhibits the protective effect of antiapoptotic Bcl-2 protein, inserting into the outer mitochondrial membrane, which in turn regulates the release of cytochrome C from the mitochondria. Cleaved caspase-3 is a protein in the caspase family that can cause an apoptotic cascade [27]. In this study, we found that cholesterol activated caspase-dependent apoptosis through mitochondrial-mediated pathways, upregulating the expression of the proapoptotic protein Bax and downregulating the expression of the antiapoptotic proteins and Bcl-xL.

In addition to apoptosis, autophagy is another key factor controlling cellular fate. Numerous evidence has revealed that autophagy plays a dual role, leading to cell death or promoting cell survival [28]. In this study, we found that cholesterol stimulation resulted in the occurrence of autophagic flux, a decrease in the protein expression of p62 and an increase in the protein expression of LC-3-II, indicating that autophagy was induced. The autophagy inhibitor 3-MA decreased the apoptosis induced by cholesterol and strengthens the inhibitory effect of cholesterol on cell viability, suggesting that cholesterol-induced autophagy may be a prosurvival mechanism.

The interaction between autophagy and apoptosis is quite complicated. Under certain circumstances, autophagy causes cell death, acting as an accessory to apoptosis. Autophagy can also promote cell growth by protecting cells from apoptotic death [29]. In this work, the presence of 3-MA significantly strengthened cholesterol-induced apoptosis. Furthermore, Z-VAD-FMK, an apoptosis inhibitor, reduced the protein expression of LC3-II and inhibited autophagic flux induced by cholesterol. These data clarified that cholesterol can induce autophagy and apoptosis in TDSCs at the same time and that apoptosis promotes autophagy, while autophagy protects TDSCs from apoptotic death. Several studies have elucidated that some proteins involved in both the apoptotic and autophagic pathways delicately regulate crosstalk, such as the Atg proteins and Bcl family proteins [30, 31]. However, the mechanisms underlying the connection between apoptosis and autophagy controlling cell survival and cell death in TDSCs are still not completely elucidated. Further study will be needed to clarify this issue.

In the present study, the induction of autophagy and apoptosis by cholesterol was accompanied by ROS generation. Overwhelming evidence has suggested that ROS mediate many signaling pathways and act as a trigger in both cell autophagy and apoptosis [32]. We found that cholesterol significantly increased the production of ROS and that the apoptosis and autophagy induced by cholesterol were blocked by NAC, indicating that cholesterol may trigger apoptosis and autophagy by inducing ROS. Previous investigations have generally suggested that ROS act as second messengers that are required for downstream signaling effects [33].

To further investigate the downstream pathways of ROS, we examined the possible pathways that mediate the processes of autophagy and apoptosis. The AKT/FOXO1 pathway has been found to regulate the processes of apoptosis and autophagy [21]. Activation of AKT protein protects cells from cell death and promotes cell survival. FOXO1, a transcription factor, is involved in a wide range of biological processes in several intracellular functions, including cell growth, proliferation, differentiation, cell cycle, and cell death. FOXO1 has also been reported to play an important role in regulating biological functions such as apoptosis and autophagy. In the present study, cholesterol decreased the active form of AKT, phosphorylated-AKT, without affecting the total protein levels of AKT. Moreover, cholesterol treatment of TDSCs led to a reduction in phosphorylated levels of FOXO1 and an increase in expression and nuclear retention of FOXO1. In addition, NAC, the ROS scavenger, markedly blocked activation of the AKT/FOXO1 pathway, suggesting that AKT/FOXO1 is a downstream pathway of ROS. Then, we confirmed that cholesterol initiated apoptosis and autophagy by the AKT/FOXO1 pathway using the FOXO1 inhibitor AS1842856. AS1842856 significantly reversed cholesterol-induced apoptosis and autophagy. It has been reported that enhanced levels of FOXO1 lead to the induction of apoptosis, which is promoted by upregulation of the expression of the proapoptotic protein BIM in several types of cell [34]. BIM is an important protein that induces apoptosis through the mitochondrial pathway by interacting with antiapoptotic proteins such as Bcl-xL. Such interactions lead to the activation of the proapoptotic protein BAX and subsequently initiate apoptosis [35]. Our findings are in agreement with these studies, demonstrating that AS1842856 reversed the upregulation of BAX and the downregulation of Bcl-xL induced by cholesterol. Iris et al. reported that FoxO1 function is essential for the maintenance of autophagic flux [36]. Among the multiple stages of autophagy, FoxO1 has been implicated in the steps of initiation, vesicle nucleation, and vesicle elongation [37, 38]. FOXO1 promotes the expression of several autophagy-related genes, such as Atg7, Atg12, and Becn1 [39]. FOXO1 upregulates the expression of autophagy genes via augmenting the expression of autophagy factors, such as Rab7 [40], and mitochondrial uncoupling proteins (UCPs). In the present study, the underlying mechanism of FOXO1-modulated autophagy needs further study.

Taken together, these results suggest that cholesterol triggers TDSC apoptosis and autophagy through ROS-activated AKT/FOXO1 signaling in vitro. In our in vivo study, the increased expression of cleaved caspase-3, Bax, LC3-II, and FOXO1 and the decreased expression of p62 in the Achilles tendon of the hypercholesterolemic group indicated that cholesterol induces apoptosis and autophagy. We have reported that CAT and NOX4 protein expression were also significantly increased in the hypercholesterolemic group [11]. Overwhelming evidence has shown that NOX4 is a source of ROS, and CAT is an antioxidant protein. Based on the results above, we surmised that high cholesterol induces apoptosis and autophagy via the ROS-mediated FOXO1 pathway in vivo.

There are a few limitations in this study. First, we did not use apoptosis inhibitors, autophagy inhibitors, antioxidants, or FOXO1 signaling inhibitors in vivo to further detect whether apoptosis, autophagy, ROS, or FOXO1 signaling might be involved in the pathogenesis of tendinopathy in hypercholesterolemia. Second, the mechanism underlying the crosstalk between apoptosis and autophagy requires further study. Thirdly, the potential mechanisms on how cholesterol induces ROS generation likely involve multiple pathways. Many previous studies have demonstrated that nicotinamide adenine dinucleotide phosphate (NADPH) oxidase, xanthine oxidase, mitochondrial enzymes, lipoxygenases, myeloperoxidases, uncoupled endothelial NO synthase, and so on are related to ROS generation under the stress of high cholesterol [41]. NADPH oxidases (NOX) are perhaps the most important ROS-generating system. Our previous experiments have showed NOX4 expression, a major isoform of NOX, was increased apparently. We would like to explore how cholesterol activates NOX4 or other kinds of oxidases directly or indirectly in the future.

## Conclusions

**Fig. 9:**
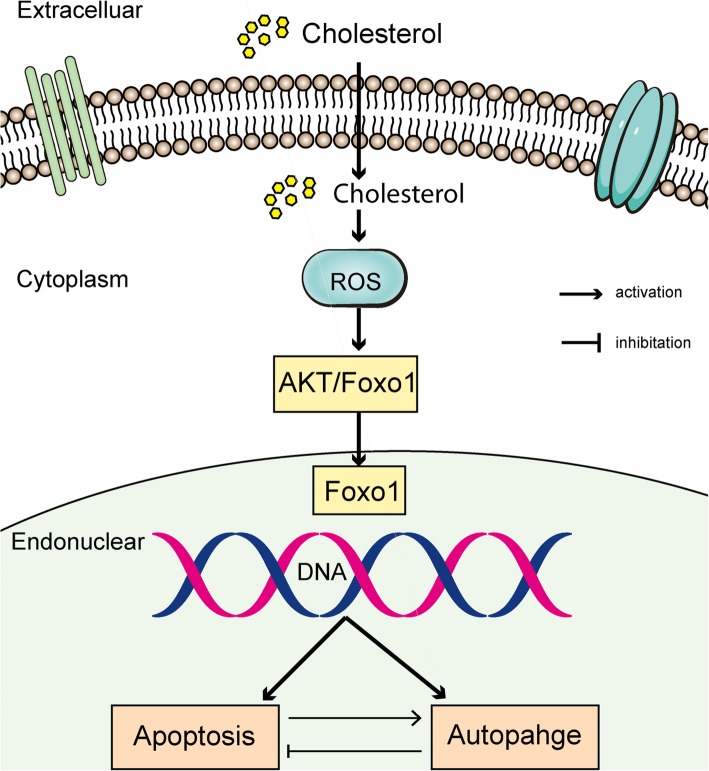
Schematic figure of the mechanism by which cholesterol induces apoptosis and autophagy in TDSCs

We have shown for the first time that high cholesterol induces apoptosis and autophagy in TDSCs. We further investigated the relationship between apoptosis and autophagy induced by cholesterol. We proposed that the lipotoxicity of cholesterol is regulated through the activation of the ROS/AKT/FOXO1 signaling pathways and that autophagy has a protective effect on TDSCs against apoptotic death (Fig. 9). Our study may contribute to the development of more effective hypercholesterolemia-induced tendinopathy therapeutic strategies, such as combination treatment with statins and autophagy inhibitors.

## Supplementary information

**Additional file 1:Supplementary Figure 1.** 3-MA inhibited autophagy induced by cholesterol. **Supplementary Figure 2.** (a, b): Z-VAD-FMK inhibited apoptosis induced by cholesterol.

## Data Availability

Please contact the corresponding author for data requests.

## Abbreviations

CHO: cholesterol
TDSCs: Tendon-derived stem cells
3-MA: 3-Methyladenine
ROS: reactive oxygen species
NAC: *N*-acetyl-l-cysteine
CCK-8: Cell Counting Kit-8 assay
APOE: Apolipoprotein E
PARP: Poly (ADPribose) polymerase

## References

[1] Wilson JJ, Best TM (2005). Common overuse tendon problems: a review and recommendations for treatment. Am Fam Physician.

[2] Dean B, Dakin SG, Millar NL, Carr AJ (2017). Review: emerging concepts in the pathogenesis of tendinopathy. Surgeon..

[3] Ozgurtas T, Yildiz C, Serdar M, Atesalp S, Kutluay T (2003). Is high concentration of serum lipids a risk factor for Achilles tendon rupture?. Clin Chim Acta.

[4] Mathiak G, Wening JV, Mathiak M, Neville LF, Jungbluth K (1999). Serum cholesterol is elevated in patients with Achilles tendon ruptures. Arch Orthop Trauma Surg.

[5] Schofield J, France M, Soran H (2013). Don’t miss familial hypercholesterolaemia in Achilles tendinopathy. BMJ..

[6] Beason DP, Abboud JA, Kuntz AF, Bassora R, Soslowsky LJ (2011). Cumulative effects of hypercholesterolemia on tendon biomechanics in a mouse model. J Orthop Res.

[7] Beason DP, Tucker JJ, Lee CS, Edelstein L, Abboud JA, Soslowsky LJ (2014). Rat rotator cuff tendon-to-bone healing properties are adversely affected by hypercholesterolemia. J Shoulder Elb Surg.

[8] Walia B, Huang AH. Tendon stem progenitor cells: understanding the biology to inform therapeutic strategies for tendon repair. J Orthop Res. 2018;37:1270–80. 10.1002/jor.24156 PMC6823601 30270569

[9] Bi Y, Ehirchiou D, Kilts TM, Inkson CA, Embree MC, Sonoyama W (2007). Identification of tendon stem/progenitor cells and the role of the extracellular matrix in their niche. Nat Med.

[10] Rui YF, Lui PP, Li G, Fu SC, Lee YW, Chan KM (2010). Isolation and characterization of multipotent rat tendon-derived stem cells. Tissue Eng Part A.

[11] Li K, Deng G, Deng Y, Chen S, Wu H, Cheng C (2019). High cholesterol inhibits tendon-related gene expressions in tendon-derived stem cells through reactive oxygen species-activated nuclear factor-kappaB signaling. J Cell Physiol.

[12] Osti L, Buda M, Del BA, Osti R, Massari L, Maffulli N (2017). Apoptosis and rotator cuff tears: scientific evidence from basic science to clinical findings. Br Med Bull.

[13] Lian O, Scott A, Engebretsen L, Bahr R, Duronio V, Khan K (2007). Excessive apoptosis in patellar tendinopathy in athletes. Am J Sports Med.

[14] Lundgreen K, Lian OB, Engebretsen L, Scott A (2011). Tenocyte apoptosis in the torn rotator cuff: a primary or secondary pathological event?. Br J Sports Med.

[15] Klionsky DJ, Emr SD (2000). Autophagy as a regulated pathway of cellular degradation. Science..

[16] Napoletano F, Baron O, Vandenabeele P, Mollereau B, Fanto M (2019). Intersections between regulated cell death and autophagy. Trends Cell Biol.

[17] Fruehauf JP, Meyskens FJ (2007). Reactive oxygen species: a breath of life or death?. Clin Cancer Res.

[18] Simon HU, Haj-Yehia A, Levi-Schaffer F (2000). Role of reactive oxygen species (ROS) in apoptosis induction. Apoptosis..

[19] Liu Q, Lei Z, Zhou K, Yu H, Liu S, Sun Q (2018). N-O reduction and ROS-mediated AKT/FOXO1 and AKT/P53 pathways are involved in growth promotion and cytotoxicity of cyadox. Chem Res Toxicol.

[20] Hemmings BA (1997). Akt signaling: linking membrane events to life and death decisions. Science..

[21] Xing YQ, Li A, Yang Y, Li XX, Zhang LN, Guo HC (2018). The regulation of FOXO1 and its role in disease progression. Life Sci.

[22] Brunet A, Bonni A, Zigmond MJ, Lin MZ, Juo P, Hu LS (1999). Akt promotes cell survival by phosphorylating and inhibiting a Forkhead transcription factor. Cell..

[23] Czabotar PE, Lessene G, Strasser A, Adams JM (2014). Control of apoptosis by the BCL-2 protein family: implications for physiology and therapy. Nat Rev Mol Cell Biol..

[24] Yang Y, Lu H, Qu J (2019). Tendon pathology in hypercholesterolaemia patients: epidemiology, pathogenesis and management. J Orthop Transl.

[25] Andarawis-Puri N, Flatow EL (2011). Tendon fatigue in response to mechanical loading. J Musculoskelet Neuronal Interact.

[26] Andarawis-Puri N, Philip A, Laudier D, Schaffler MB, Flatow EL (2014). Temporal effect of in vivo tendon fatigue loading on the apoptotic response explained in the context of number of fatigue loading cycles and initial damage parameters. J Orthop Res.

[27] Zakeri Z, Bursch W, Tenniswood M, Lockshin RA (1995). Cell death: programmed, apoptosis, necrosis, or other?. Cell Death Differ.

[28] Singh SS, Vats S, Chia AY, Tan TZ, Deng S, Ong MS (2018). Dual role of autophagy in hallmarks of cancer. Oncogene..

[29] Song S, Tan J, Miao Y, Li M, Zhang Q (2017). Crosstalk of autophagy and apoptosis: involvement of the dual role of autophagy under ER stress. J Cell Physiol.

[30] Eisenberg-Lerner A, Bialik S, Simon HU, Kimchi A (2009). Life and death partners: apoptosis, autophagy and the cross-talk between them. Cell Death Differ.

[31] Mukhopadhyay S, Panda PK, Sinha N, Das DN, Bhutia SK (2014). Autophagy and apoptosis: where do they meet?. Apoptosis..

[32] Ling LU, Tan KB, Lin H, Chiu GN (2011). The role of reactive oxygen species and autophagy in safingol-induced cell death. Cell Death Dis.

[33] Sies H, Berndt C, Jones DP (2017). Oxidative stress. Annu Rev Biochem.

[34] Wang Y, Tang H, He G, Shi Y, Kang X, Lyu J (2018). High concentration of aspirin induces apoptosis in rat tendon stem cells via inhibition of the Wnt/beta-catenin pathway. Cell Physiol Biochem.

[35] Strasser A, Puthalakath H, Bouillet P, Huang DC, O'Connor L, O'Reilly LA (2000). The role of bim, a proapoptotic BH3-only member of the Bcl-2 family in cell-death control. Ann N Y Acad Sci.

[36] Schaffner I, Minakaki G, Khan MA, Balta EA, Schlotzer-Schrehardt U, Schwarz TJ (2018). FoxO function is essential for maintenance of autophagic flux and neuronal morphogenesis in adult neurogenesis. Neuron..

[37] Webb AE, Brunet A (2014). FOXO transcription factors: key regulators of cellular quality control. Trends Biochem Sci.

[38] Fullgrabe J, Klionsky DJ, Joseph B (2014). The return of the nucleus: transcriptional and epigenetic control of autophagy. Nat Rev Mol Cell Biol.

[39] Shen M, Cao Y, Jiang Y, Wei Y, Liu H (2018). Melatonin protects mouse granulosa cells against oxidative damage by inhibiting FOXO1-mediated autophagy: implication of an antioxidation-independent mechanism. Redox Biol.

[40] Wang B, Ding W, Zhang M, Li H, Guo H, Lin L (2016). Role of FOXO1 in aldosterone-induced autophagy: a compensatory protective mechanism related to podocyte injury. Oncotarget..

[41] Kattoor AJ, Pothineni N, Palagiri D, Mehta JL (2017). Oxidative stress in atherosclerosis. Curr Atheroscler Rep.

