# Supplementary material for: High cholesterol induces apoptosis and autophagy through the ROS-activated AKT/FOXO1 pathway in tendon-derived stem cells
Source: Stem Cell Res Ther. 2020 Mar 20;11:131. doi: 10.1186/s13287-020-01643-5 (PMC7082977; doi:10.1186/s13287-020-01643-5)
Supplement: Supplementary file 1 — Additional file 1:Supplementary Figure 1. 3-MA inhibited autophagy induced by cholesterol. Supplementary Figure 2. (a, b): Z-VAD-FMK inhibited apoptosis induced by cholesterol. [file 13287_2020_1643_MOESM1_ESM.docx]

**Supplementary figure and legends:**


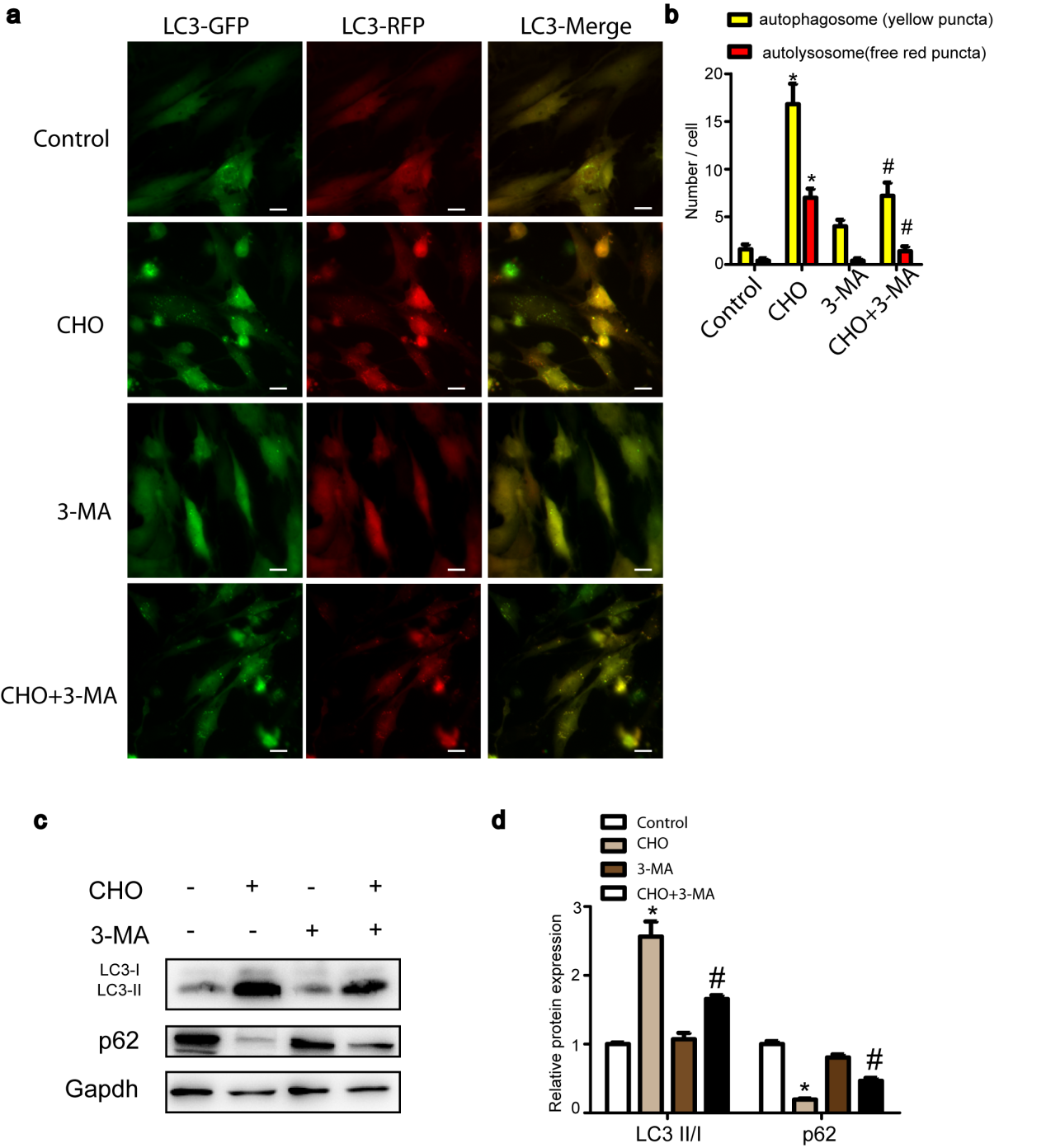


**Supplementary Fig.1: 3-MA inhibited autophagy induced by cholesterol.** (a, b): Cells were transfected with the mRFP-GFP-LC3 vector for 24 h and then pretreated with 3-MA (1 mM, 1 h) before incubation with 10 mg/dL cholesterol and 3-MA for 24 h. Representative images of fluorescent LC3 puncta are shown. Bar: 20 µm. (c, d): TDSCs were pretreated with 3-MA (1 mM, 1 h) before incubation with 10 mg/dL cholesterol and 3-MA for 24 h. Autophagy-related proteins (LC3-II and p62) were analyzed by western blotting. All quantitative data are expressed as the means ± SEM of the results from three independent experiments. ∗ p <0.05 versus control, # p <0.05 versus CHO. CHO: cholesterol.


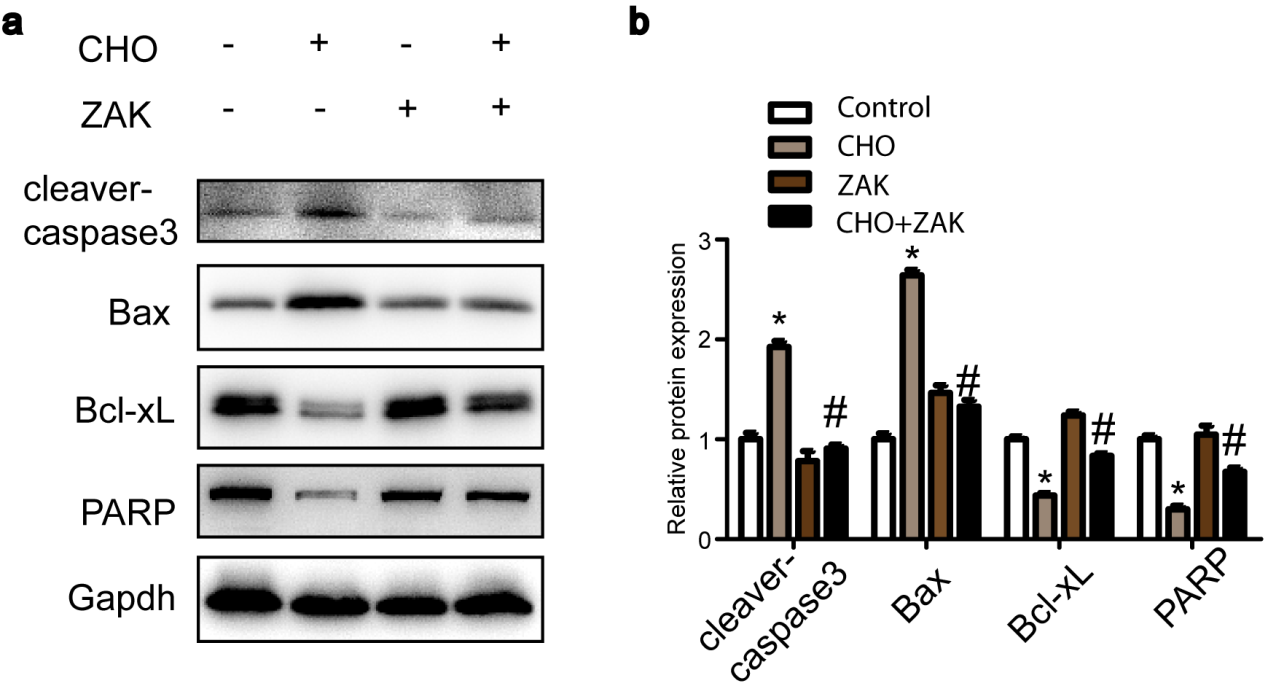


**Supplementary Fig.2: (a, b): Z-VAD-FMK inhibited apoptosis induced by cholesterol.** TDSCs were pretreated with Z-VAD-FMK (20 µM, 1 h) before incubation with 10 mg/dL cholesterol and Z-VAD-FMK for 24 h. Apoptosis-related proteins (cleaved caspase-3, Bax, Bcl-xL, and PARP) were analyzed by western blotting. All quantitative data are expressed as the means ± SEM of the results from three independent experiments. ∗ p <0.05 versus control, # p <0.05 versus CHO. CHO: cholesterol. ZAK: Z-VAD-FMK.
